# Transmission of SARS-CoV-2 Omicron Variant under a Dynamic Clearance Strategy in Shandong, China

**DOI:** 10.1128/spectrum.04632-22

**Published:** 2023-03-14

**Authors:** Yifei Xu, Ti Liu, Yan Li, Xuemin Wei, Zhaoguo Wang, Ming Fang, Yuwei Zhang, Huaning Zhang, Lifang Zhang, Jinbo Zhang, Jin Xu, Yunlong Tian, Nianzheng He, Yuhan Zhang, Yao Wang, Mingxiao Yao, Bo Pang, Shuang Wang, Hongling Wen, Zengqiang Kou

**Affiliations:** a Department of Microbiology, School of Public Health, Cheeloo College of Medicine, Shandong University, Jinan, Shandong, China; b Shandong Center for Disease Control and Prevention, Jinan, Shandong, China; c Qingdao Center for Disease Control and Prevention, Qingdao, Shandong, China; d Binzhou Center for Disease Control and Prevention, Binzhou, Shandong, China; e Weihai Center for Disease Control and Prevention, Weihai, Shandong, China; f Zibo Center for Disease Control and Prevention, Zibo, Shandong, China; g Yantai Center for Disease Control and Prevention, Yantai, Shandong, China; h Suzhou Research Institute of Shandong University, Suzhou, Jiangsu, China; University of Siena

**Keywords:** SARS-CoV-2, Omicron variant, genomic epidemiology, phylogenetic analysis, transmission

## Abstract

SARS-CoV-2 Omicron caused a large wave of COVID-19 cases in China in spring 2022. Shandong was one of the most affected regions during this epidemic yet was also among those areas that were able to quickly contain the transmission. We aimed to investigate the origin, genetic diversity, and transmission patterns of the Omicron epidemic in Shandong under a dynamic clearance strategy. We generated 1,149 Omicron sequences, performed phylogenetic analysis, and interpreted results in the context of available epidemiological information. We observed that there were multiple introductions of distinct Omicron sublineages into Shandong from foreign countries and other regions in China, while a small number of introductions led to majority of local cases. We found evidence suggesting that some local clusters were potentially associated with foreign imported cases. Superspreading events and cryptic transmissions contributed to the rapid spread of this epidemic. We identified a BA.1.1 genome with the R493Q reversion mutation in the spike receptor binding domain, potentially associated with an escape from vaccine and Omicron infection elicited neutralizing immunity. Our findings illustrated how the dynamic clearance strategy constrained this epidemic's size, duration, and geographical distribution.

**IMPORTANCE** Starting in March 2022, the Omicron epidemic caused a large wave of COVID-19 cases in China. Shandong was one of the most affected regions during this epidemic but was also among those areas that were able to quickly contain the transmission. We investigated the origin, genetic diversity, and transmission patterns of Omicron epidemic in Shandong under a dynamic clearance strategy. We found that there were multiple introductions of distinct Omicron sublineages into Shandong from foreign countries and other regions in China, while a small number of introductions led to most local cases. We found evidence suggesting that some local clusters were associated with foreign imported cases. Superspreading events and cryptic transmissions contributed to the rapid spread of this epidemic. Our study illustrated the transmission patterns of Omicron epidemic in Shandong and provided a looking glass onto this epidemic in China.

## INTRODUCTION

Since the onset of COVID-19 pandemic, the world has witnessed repeated emergence of severe acute respiratory coronavirus 2 (SARS-CoV-2) variants (1–3). The World Health Organization has designated several variants as variants of concern (VOCs) due to their increased risks to global public health. The Omicron VOC, identified in November 2021, has become the dominant strain globally since the beginning of 2022 (4). The Omicron variant is characterized as the most transmissible but the least pathogenic among all existing SARS-CoV-2 variants (5–8).

China has adopted a dynamic clearance strategy to stamp out SARS-CoV-2 outbreaks through implementation of control measures, such as stringent border control, meticulous quarantine, and mass screening (9). This strategy has been proven to be effective in containing the previous VOCs and maintaining an extended period of low level of SARS-CoV-2 cases (10–12). Omicron was the first variant imported into China in December 2021. Since March 2022, the Omicron epidemic has caused the largest spike in COVID-19 cases in China after the outbreak of this pandemic in Wuhan, December 2019. As of 30 May 2022, the Omicron epidemic has spread to 31 provinces and resulted in more than 750,000 confirmed cases in China. This epidemic has forced the imposition of lockdown in several major cities, including Shanghai and Shenzhen.

Shandong Province, located at east China, has a large population of more than 100 million people. It was one of the most affected regions in this Omicron epidemic, but also among those that were able to contain the transmission. Here, we report the genomic epidemiology of the Omicron epidemic in Shandong. We recovered over 1,000 Omicron genomic sequences, performed phylogenetic analysis, and interpreted results in the context of available epidemiological information. Our objectives were to investigate the origin, genetic diversity, and transmission patterns of this Omicron epidemic in Shandong, and understand how the dynamic clearance strategy was contributing to the reduction of transmission. Findings in this study provided relevant information for interpreting genomic epidemiology of Omicron epidemic in other regions in China.

## RESULTS

### Genomic sequencing of Omicron in Shandong.

**FIG 1:**
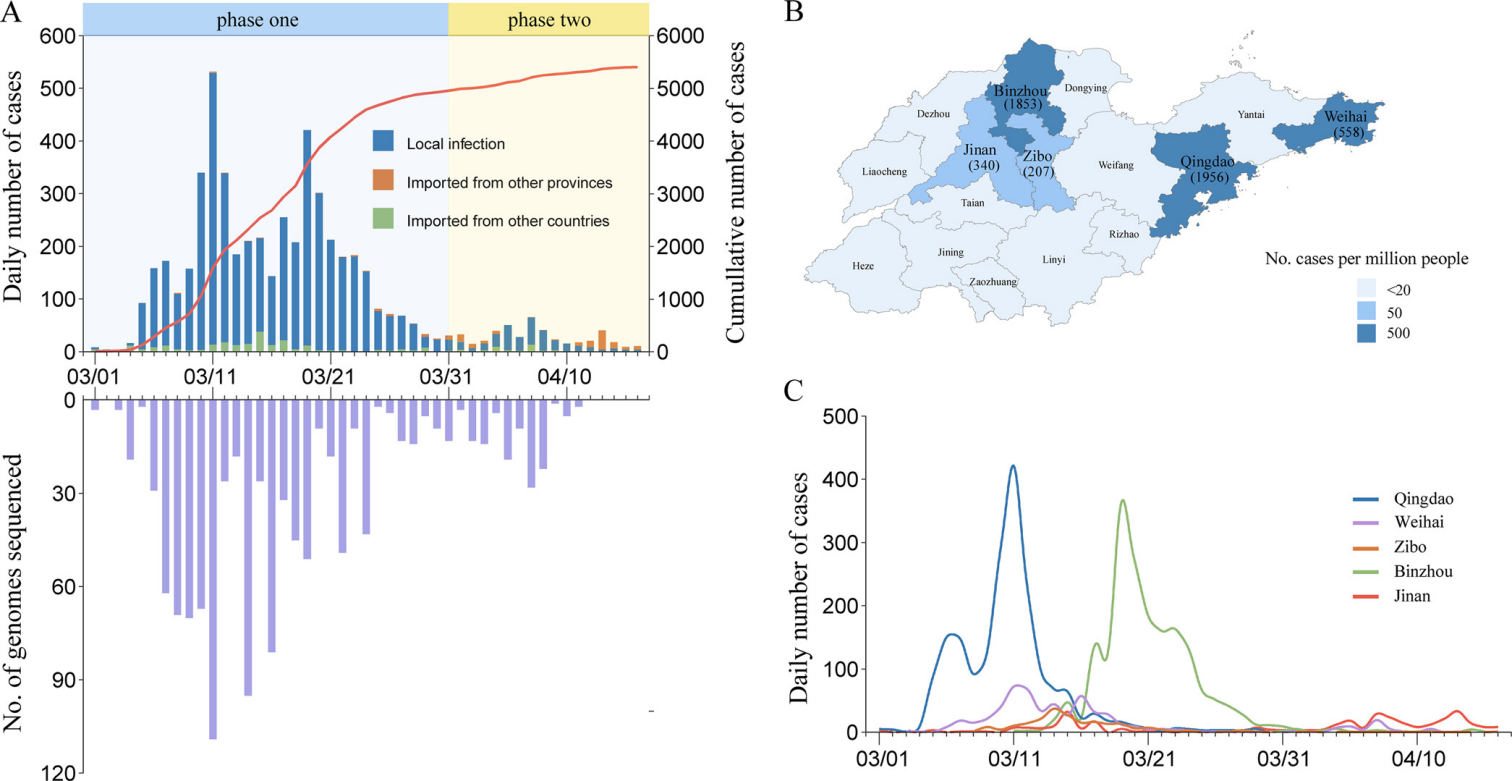
Number and distribution of SARS-CoV-2 cases and genomes from the Omicron epidemic in Shandong, China. (A) Number of laboratory-confirmed SARS-CoV-2 cases in Shandong by reported date. The cases are classified as local infection, imported from other regions in China, and imported from other countries. The cumulative number of cases is shown by a red line. The number of genomic sequences recovered from this study is shown over time. (B) Map of Shandong Province shaded by the number of laboratory-confirmed SARS-CoV-2 cases per million people. Number of laboratory-confirmed SARS-CoV-2 cases are shown for five cities with the largest number of infections. (C) Time series of laboratory-confirmed SARS-CoV-2 cases for five cities with the largest number of infections. Map obtained from Resource and Environment Science and Data Center (http://www.resdc.cn/data.aspx?DATAID=201).

We identified the first SARS-CoV-2 case in Shandong on 1 March 2022 (Fig. 1A). Since then, the daily case count rapidly rose to its peak within 10 days. By 16 April, the total number of confirmed cases reached 5,404. Among them, there were 4,993 (92.3%) local cases, 152 (2.8%) domestic imported cases from other regions in China, and 259 (4.7%) foreign imported cases from other countries. This epidemic spread to 16 cities in Shandong (Fig. 1B). Five cities represented 90.9% of the total cases, including Qingdao (1,956; 36.1%), Binzhou (1,853; 34.2%), Weihai (558; 10.3%), Jinan (340; 6.2%), and Zibo (207; 3.8%) (Fig. 1B and C).

We performed sequencing for SARS-CoV-2 PCR-positive samples collected during this epidemic and successfully recovered 1,115 SARS-CoV-2 genomic sequences with >90% coverage from 1,115 individuals (Fig. 1A), representing 20.7% of total confirmed cases. In addition, we retrieved 34 SARS-CoV-2 genomic sequences from foreign imported cases in Shandong between January and February 2022. Our final data set contained a collection of 1,149 SARS-CoV-2 genomic sequences.

### Multiple introductions of Omicron into Shandong.

Shandong has maintained an extended period of zero reported SARS-CoV-2 cases before March 2022. It is of great scientific interest that we sought to understand the origin of this Omicron epidemic. Our main task is to answer whether it is caused by recent introductions from other regions or by resurgence of local strains that were not previously identified.

We first determined that all the genomes in this epidemic were Omicron variants. Then, we studied the continuous introductions of distinct Omicron lineages into Shandong by foreign and domestic imported cases. International travelers entered Shandong via three port cities, Qingdao, Weihai, and Yantai, all of which operated air flights connecting Shandong, South Korean and Japanese cities. The foreign imported genomes represented seven distinct genetic sublineages and were primarily from BA.1.1, BA.2, and BA.2.3 between January and March 2022.

While the epidemic spread across China, Shanghai Municipality and Jilin Province had become the centers of this Omicron epidemic since mid-March. We followed this trail of domestic imported cases, which started to present at a higher proportion in the Shandong daily case count since the end of March. In contrast to the greater genetic diversity of foreign imported genomes, the domestic imported genomes were from two sublineages. Those from Shanghai were BA.2.2, while others from Jilin were BA.2. The Shandong local genomes were primarily in sublineages of BA.1.1, BA.2, BA.2.2, and BA.2.3, which fall into the spectrum of both the foreign and domestic imported genomes.

**FIG 2:**
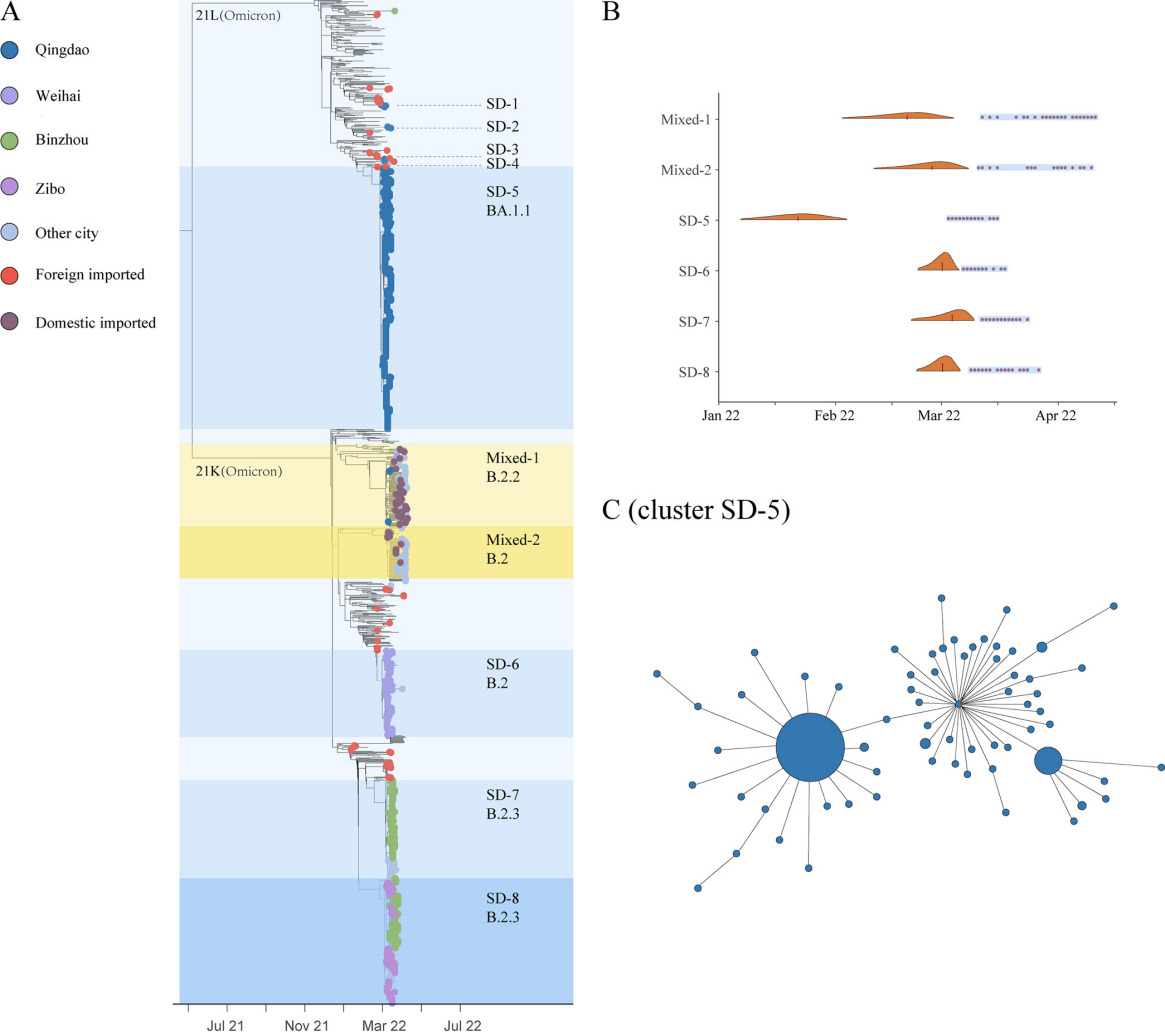
Phylogenetic tree of SARS-CoV-2 genomic sequences recovered from the Omicron epidemic in Shandong, China. (A) Maximum-likelihood time-scaled phylogenetic tree of SARS-CoV-2 genomes from this study and reference genomes representing global SARS-CoV-2 genetic diversity. Shandong Omicron genomes are denoted by small circle and colored according to the sampling city, domestic introduction from other regions in China, and foreign introduction from other countries. Cluster assignment and genetic clades are indicated on the right of the tree. (B) Violin plots of TMRCA for the major clusters identified in this study. Distributions are truncated at the upper and lower limits of the 95% HPD intervals. The blue shading denotes the genomic sampling period for each cluster. Dark red dots indicate the sampling date for each genome. (C) Minimum spanning tree showing genetic similarity of genomes within cluster SD-5. The size of the circle reflects the number of identical genomes. The largest circle denotes 274 identical BA.1.1 genomic sequences. Genomes within cluster SD-5 differed from one another between 0 and 6 SNPs.

To further investigate the genetic relationship between local and imported genomes, we conducted phylogenetic analysis with genomes in our collection and reference genomes representing global SARS-CoV-2 genetic diversity. We analyzed the phylogeny together with the public health investigation results. The foreign imported Omicron genomes distributed across the phylogenetic tree and largely reflected the global genetic diversity (Fig. 2A). The domestic imported and local genomes could be classified into 10 clusters with two distinct patterns. Cluster SD-1 to SD-8 contained Shandong local genomes in March, while cluster Mixed-1 and Mixed-2 both contained a mixture of domestic imported and local genomes in April (Fig. 2A). These eight local clusters were in divergent monophyletic clades and from three genetic sublineages, BA.1.1 (SD-1 to SD-5), BA.2 (SD-6), and BA.2.3 (SD-7 and SD-8). Each cluster is corresponding to genomes primarily from one or two cities rather than wide-spread transmission across Shandong, which likely reflected the effectiveness of the stringent travel restriction and contact tracing implemented under the dynamic clearance strategy. These observations, together with public health investigation, indicated that this epidemic was caused by multiple independent introductions into Shandong.

China has implemented stringent border control under the dynamic clearance strategy. All international travelers entering China were requested to take a pretravel molecular test, receive a test by the customs authorities, and subject to a 14-day quarantine in the first entry point city. We found evidence suggesting that some local clusters could potentially be associated with foreign imported case. Cluster SD-1 was comprised of six BA.1.1 sequences that were recovered from patients in Qingdao. This cluster included the first known patient of this epidemic in Shandong. The patient was a bus driver and tested positive for SARS-CoV-2 on 1 March 2022. In the following week, five family members and colleagues of the first patient were tested positive. Public health investigation showed that these patients had no recent travel history outside Qingdao. In the phylogeny, cluster SD-1 was most closely related to a sequence that was recovered from an international traveler on 27 February 2022 in the same city. Comparison showed no single-nucleotide polymorphism (SNP) difference between the foreign imported genome and the six local genomes in cluster SD-1. Although the exact source of infection and transmission route cannot be determined for cases in cluster SD-1, our findings indicated potential linkage between foreign Omicron introductions and local cases in Shandong.

**FIG 3:**
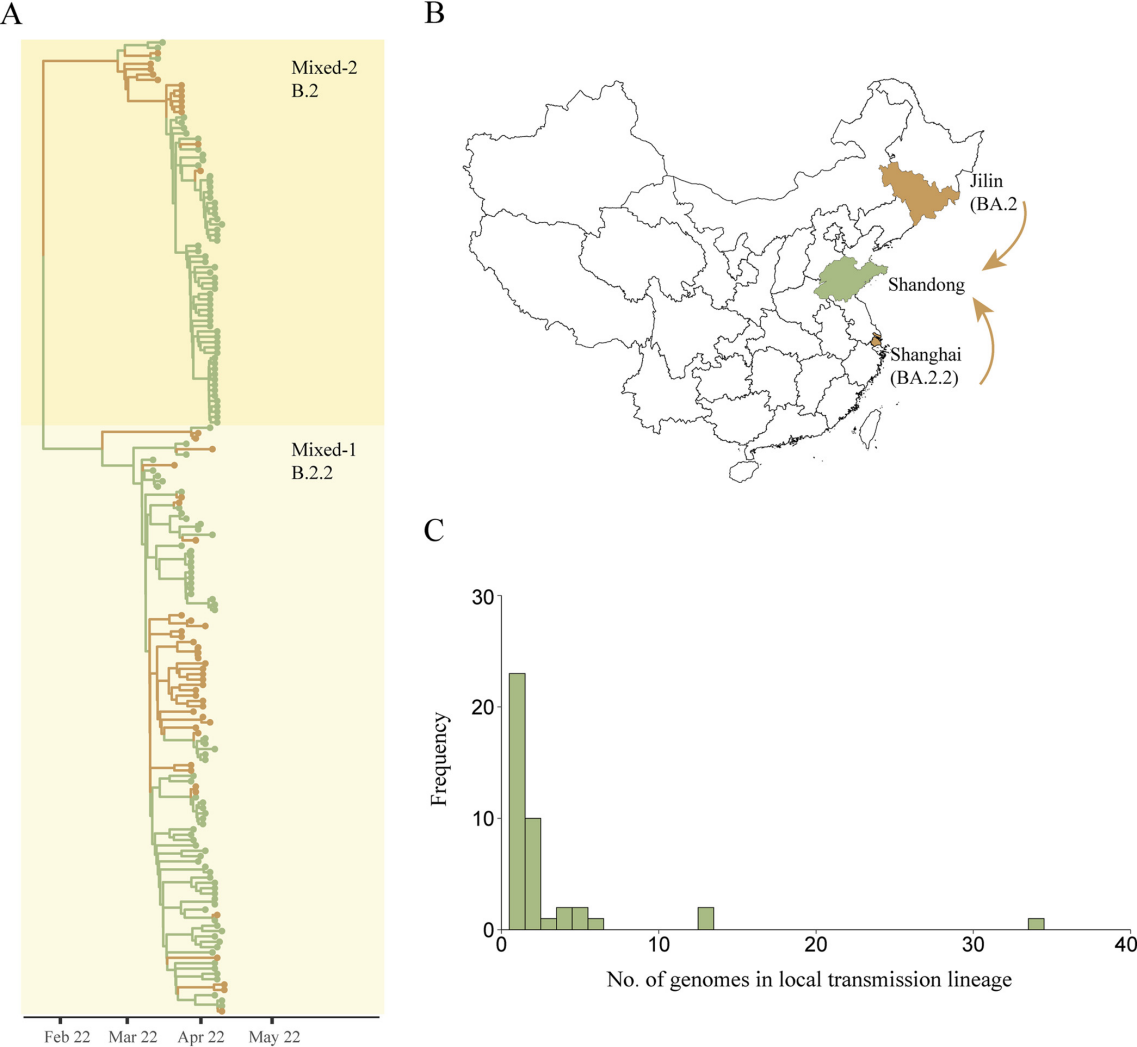
Importation of SARS-CoV-2 from other regions in China and associated local transmissions in Shandong. (A) Maximum clade credibility time-scaled phylogenetic tree of domestic imported cases from other regions in China and local cases in Shandong. Domestic imported cases and local cases are colored by yellow and green, respectively. Shanghai Municipality and Jilin Province have become the centers of this Omicron epidemic in China since mid-March 2022. The BA.2.2 genomes in cluster Mixed-1 were primarily imported from Shanghai, while the BA.2 genomes in cluster Mixed-2 were primarily imported from Jilin. (B) Map of China showing Shandong Province, Shanghai Municipality and Jilin Province. (C) Frequency of the number of genomes in each local transmission lineage in Shandong. Map obtained from Resource and Environment Science and Data Center (http://www.resdc.cn/data.aspx?DATAID=201).

Cluster Mixed-1 and Mixed-2 contained a mixture of domestic imported genomes and local genomes from Shandong in April (Fig. 2A and Fig. 3A). The BA.2.2 domestic imported genomes in cluster Mixed-1 were primarily from Shanghai, while the BA.2 genomes in cluster Mixed-2 were primarily imported from Jilin. These results revealed that the dominant Omicron lineage circulating in Shanghai and Jilin were BA.2.2 and BA.2, respectively (Fig. 3A and B). Public health investigation found that multiple domestic introductions of BA.2.2 and BA.2 have not caused large case clusters in Shandong. Through phylogenetic analysis and ancestral trait reconstruction, we identified that 23 domestic introductions in cluster Mixed-1 and Mixed-2 led to only one other secondary case in Shandong, while 19 introductions led to ongoing transmission with a median size of only 2 genomes (Fig. 3C).

Cluster Mixed-1 was most closely related to genomes originated from Hongkong between January and March 2022 in the phylogeny, indicating the explosive spreading of Omicron BA.2.2 in Shanghai could be associated with foreign imported cases. We estimated that the median time to most recent common ancestor (TMRCA) for cluster Mixed-1 was 19 February 2022 (95% highest posterior density [HPD] 2 February to 4 March) (Fig. 2B). These findings were consistent with previous report that the source of infection in Shanghai was from a designated quarantine hotel for inbound foreign travelers. Our TMRCA estimate coincided with the time window when this hotel started to serve as a quarantine site for inbound travelers on 16 February 2022. In addition, we found that cluster Mixed-1 and Mixed-2 were diverse with respect to geographic sampling, containing genomes from across multiple regions in China. This finding indicated the widespread transmission of the Omicron BA.2.2 and BA.2 across China.

### Local transmission cluster.

Local case clusters began to appear soon after detection of this Omicron epidemic in Shandong. We focused our investigation on the largest case cluster, which was recognized in the context of a school in Qingdao on 5 March 2022. Students, teachers, and their family members were soon tested positive for SARS-CoV-2 by PCR. Public health investigation with contact tracing showed that at least 1,208 cases were epidemiologically linked to that school. This raised the question whether a superspreading event had occurred there. Therefore, we sequenced 398 Omicron BA.1.1 genomes from cases either epidemiologically linked to that school or detected within the same time window in Qingdao. Phylogenetic analysis showed that these genomes formed a very tight cluster (SD-5) with limited genetic variation (Fig. 2A and C). They differed from one another by a median of only one SNP (range 0 to 6 SNPs). In addition, we found that 274 genomes were identical, representing 68.8% of genomes within cluster SD-5 (Fig. 2C). The presence of large number of highly similar genomes within a short time window, in combination with the public health investigation data, suggested that it was a superspreading event that have contributed to the rapid amplification of the largest case cluster in this epidemic.

We also investigated key amino acid mutations with known functional significance. Genomic characterization showed that our sequences carried a few mutations in the viral spike protein that were known to increase transmissibility, such as Q498R, N501Y, H655Y, N679K, and P681H. Q493R mutation in the spike receptor binding domain is another defining signatures of Omicron genomic sequences. However, one of our BA.1.1 sequence in cluster SD-5 harbored the R493Q reversion mutation. The 493 residue lies within the ACE2 footprint, therefore has the potential to modulate ACE2 affinity and the neutralizing capacity of vaccine or naturally acquired serum (13). This R493Q reversion is also found in the emerging Omicron BA.4 and BA.5 sublineages, which show escape from vaccine and BA.1 infection elicited neutralizing immunity (14, 15). BA.4 and BA.5 have been growing in prevalence in South Africa since the beginning of 2022 and may spread globally to drive another wave of infections (16–18).

We estimated that the median TMRCA for cluster SD-5 was 21 January 2022 (95% HPD 6 January to 4 February) (Fig. 2B). The detection lag (defined as the number of days between the median TMRCA and the earliest sequence collection date in the cluster) was over 1 month. These results suggested potential prolonged cryptic transmission preceded the detection of cluster SD-5. SD-5 was the first large case cluster identified in this epidemic. We estimated TMRCA for three latter clusters for comparison. The estimated median TMRCA for SD-6, SD-7, and SD-8 were in the first week of March 2022. The detection lags for these three clusters were between 6 and 9 days, and the number of days between the median TMRCA and the earliest disease onset date in each cluster was 6 days, reflecting improvements in case detection in this epidemic.

## DISCUSSION

The Omicron variant has caused the largest outbreak in China since the emergence of this pandemic 2 years ago, presenting a great challenge to the dynamic clearance strategy. In this study, we generated over 1,000 Omicron genomes from infected individuals in Shandong. Our data revealed that there were continuous introductions of distinct Omicron sublineages into Shandong from foreign countries and other regions in China. Foreign imported cases were primarily in BA.1.1, BA.2, and BA.2.3, while domestic imported cases from Shanghai and Jilin were in BA.2.2 and BA.2. Only a small number of introductions out of multiple introductions dominated the epidemic and led to most of the cases. Similar patterns were observed by previous studies of SARS-CoV-2 in the United States (19, 20).

Our study found evidence suggesting that some local transmission clusters in Shandong were likely initiated by foreign imported cases. Under China’s dynamic clearance strategy, all inbound international travelers were requested to take a 2-week mandatory quarantine in the first entry point city. Breaches in the screening and quarantine of inbound travelers from high-risk regions have caused major outbreaks. In Hongkong, an incident in a quarantine hotel was the source of infection for the large outbreak of Omicron BA.2 in early 2022 (21, 22). Transmission first occurred in the quarantine hotel, and later caused large scale community transmission. The explosive transmission of Omicron BA.2 in Shanghai was also due to failures in infection control measures in a designated quarantine hotel for inbound international travelers (23). While our data did not allow us to determine the potential origin and transmission pathway for each domestic transmission cluster in Shandong, six out of eight domestic clusters were from three port cities, Qingdao, Weihai, and Yantai. International travelers from South Korean and Japanese cities landed in these cities. Our findings indicated potential transmission in quarantine hotel and subsequent community transmission despite strict quarantine precautions.

Shandong had zero reported SARS-CoV-2 cases for an extended period before March 2022. Genomic surveillance for foreign imported SARS-CoV-2 cases were important for inferencing the origin of domestic transmission. Our data showed that all SARS-CoV-2 genomes recovered in this study were Omicron and shifting from BA.1 to BA.2 in this Shandong epidemic. A similar trend was observed in a study using all foreign imported cases into China (24). In addition, this was in accordance with the global spread of Omicron in the first half of 2022. All Omicron genomes from this study belong to the existing Pango lineages, and no novel genetic lineages were detected.

The Omicron variant is different from previous variants in terms of transmissibility and pathogenicity (5, 25, 26). Asymptomatic or mild symptoms presented by infected individuals make it challenging for the timely identification of Omicron infection. Among all the cases during this Shandong epidemic, over 96% cases were either asymptomatic or presented mild symptoms. Estimation of TMRCA suggested prolonged cryptic transmission preceded the detection of the largest case cluster in this epidemic. In addition, the largest case cluster was amplified by the superspreading event in a school. Superspreading and cryptic transmission together contributed to the spread of Omicron variant in Shandong.

The spike protein of the Omicron variant is characterized by 34 mutations (27, 28). The Q493R amino acid mutation in the spike receptor binding domain has been associated with resistance to bamlanivimab and etesevimab (29, 30). A previous study has reported the emergence of Q493R mutation in a patient who was treated with these two drugs (29). In addition, the R493Q reversion mutation now occurs in the emerging Omicron BA.4 and BA.5 sublineages, which are the new variants of concern and may fuel increase in global infection. Both BA.4 and BA.5 contained the L452R, F486V, and R493Q mutations compared to BA.2 (17). Preliminary studies showed that these mutations have caused a significant change in the antigenic properties of BA.4 and BA.5 compared to BA.1 and BA.2. We also identified the R493Q reversion mutation in one of our BA.1.1 genome, highlighting the need to study the genesis, prevalence, and clinical impact of the R493Q mutation.

Dynamic clearance strategy was the general guideline in China’s fight against COVID-19. This strategy was introduced to fight the Delta variant in August 2021 and the subsequent highly transmissible Omicron variant in 2022. The aim of this strategy was to minimize the impact of the epidemic on the society and people’s normal lives through quick identification and containment of outbreaks (31). Precise prevention and control measures were crucial for the success of this strategy. Nucleic acid screening has been widely used in China to quickly detect infections in the populations. Big data technology has been applied to trace close contacts and risk groups, allowing rapid interventions of transmission. Personal protective measures, such as wearing masks and social distancing, have been encouraged to protect susceptible population. A modeling study estimated that Omicron could cause over 1.5 million deaths in China with current vaccine coverage and access to antiviral therapies (32). It is also important to note that the dynamic clearance strategy was established based on lessons China learned from COVID-19. Country or region-specific infection prevention and control strategies based on its own COVID-19 epidemic situation, health resources, and response capacity are needed.

Shandong has taken a series of infection control measures to contain this Omicron epidemic. Multiple rounds of city-wide SARS-CoV-2 nucleic acid screening have been conducted in cities that were mostly affected. Moreover, school, restaurants, construction sites, and other high-risk places were closed. The capital city Jinan had undergone partial lock down for multiple days during this epidemic. Furthermore, all domestic travelers entered Shandong were requested to take a molecular test pretravel and after arrival. Travelers from high-risk regions were subjected to quarantine and medical observations. These measures collectively contributed to the transmission reduction of this Shandong Omicron epidemic on the province level within a month.

Genome sequencing can provide high resolution characterization of the spatiotemporal spread of viral outbreaks (33, 34). The sequencing data generated in this study allowed us to identify transmission clusters and unveil transmission patterns of the Omicron epidemic in Shandong. Some large transmission clusters contained a large fraction of identical consensus genomic sequences, which limited our ability to infer transmission links between infected individuals. In addition, understanding the exact source and transmission route of this Omicron epidemic has been impeded by the very limited number of public available Omicron genomic sequences from other regions in China and the gaps in the global record of available genomes.

While the Omicron epidemic continues in China, our data illustrated its transmission patterns in Shandong and provided a looking glass onto this epidemic under the dynamic clearance strategy. Our study demonstrated that genome-based surveillance can provide timely and precise insights for transmission dynamics of viral outbreaks, inform public health decision making, and contribute to infection control practices.

## MATERIALS AND METHODS

### Ethical statement and data collection.

This study was conducted under approval from Shandong University and ethical approval from the Center for Disease Control and Prevention of Shandong Province. Samples testing positive for SARS-CoV-2 by real-time PCR were obtained from public health diagnostics laboratories locate throughout Shandong and sequenced as part of routine public health surveillance activities. Public health investigations were conducted by Shandong CDC and other city CDC. The data included report date, patients’ general personal information, contact history, and potential exposures. Data were anonymized before the analysis.

### Nucleic acid testing.

Real-time reverse transcription-PCR (rRT-PCR) testing for SARS-CoV-2 was performed according to guidelines from China CDC. The testing targets the open reding frame 1ab (ORF1ab) and the nucleocapsid protein (N) gene of SARS-CoV-2 genome. Viral RNA was extracted, and rRT-PCR was performed using the SARS-CoV-2 detection kit (BioGerm Medical Biotechnology Co., Ltd.) according to the manufacturer’s instructions.

### SARS-CoV-2 genome sequencing.

Viral RNAs were extracted from samples using QIAamp Viral RNA minikit (Qiagen, Inc., Hilden, Germany). The RNA was reverse transcribed and amplified using ULSEN 2019-nCoV whole-genome capture kit (Beijing MicroFuture Technology Co., Ltd., Beijing, China), followed by purification with a 1:1 ratio of AMPure XP beads (Beckman Coulter, Inc., Brea, CA, US). Purified cDNA was quantified using Qubit 1× dsDNA high sensitivity (HS) assay kits (Thermo Fisher Scientific, Inc., Waltham, US). The sequencing libraries were prepared using purified cDNA at a concentration of 0.2 ng/μL, Nextera XT library prep kit and Nextera XT index kit (Illumina, Inc., San Diego, CA, US). Sequencing was conducted using Illumina Nextseq2000 or MiSeq platform.

### Genome assembly and analysis.

The sequencing data were analyzed using an in-house bioinformatics pipeline. The SARS-CoV-2 consensus sequences were generated using a reference-based assembly approach. Briefly, sequencing reads were mapped to the reference genome (Wuhan-Hu-1, NCBI NC_045512.2) using BWA-MEM v0.7.17 (35). Consensus sequences were generated with a minimum 10-fold mapping coverage and supported by at least 70% of reads at a given position. The mapping profile was visualized using the Integrative Genomics Viewer and checked to correct potential assembly errors. Consensus sequences less than 27 kb in length were excluded from downstream analysis. If multiple SARS-CoV-2 positive samples were collected and sequenced for the same infected individual, the most complete genome was kept for the analysis. The genetic clade for each SARS-CoV-2 consensus sequence was determined using Nextclade v1.11.0 (https://clades.nextstrain.org).

### Phylogenetic analysis.

Phylogenetic analysis was performed using an integrated data set comprised of SARS-CoV-2 genomes from this study and a reference set of publicly available sequences from GISAID (https://gisaid.org/; accessed April 2022). We downloaded the representativity global genome data set and six regional-specific genome data sets from GISAID. For the preliminary phylogenic analysis, we included: (i) Shandong Omicron genomes from this study; (ii) GISAID global representativity genomes; (iii) all Omicron genomes originated from China; and (iv) the top three BLAST hits when querying Shandong Omicron genomic sequences against the global and six regional-specific representativity genome data sets. These data set comprised a collection of 3,796 genomic sequences. Sequence alignment was performed using MAFFT v7.310 (36). The untranslated regions (UTRs) from both ends of the genomes (first 265 and last 228 bases) were trimmed before phylogenetic analysis. A preliminary maximum-likelihood (ML) phylogenetic tree was generated using IQ-TREE v2.0.3 (37), implementing the best-fit nucleotide substitution model determined by the program and NC_045512.2 as an outgroup root. Based on the preliminary ML phylogenetic tree, 1,526 sequences were selected for more detailed phylogenetic and molecular clock analyses. Large clades in the preliminary tree that contained only reference genomes were pruned to three representatives per clade. The final phylogenetic tree was built using IQ-TREE v2.0.3 (37), with branch support by ultrafast bootstrap approximation and SH-like approximate likelihood ratio test. Inference of divergence times was performed using TreeTime v2.0.3 (38). The phylogenetic tree was visualized using R package ggtree v3.2.1 (39).

TMRCA was estimated using the Bayesian Markov chain Monte Carlo method implemented in BEAST v1.10.4 (40, 41). The temporal signal of the data set was investigated using a root-to-tip regression of genetic distances against sampling date in TempEst v1.5 (42). Outliers were removed before the analysis. HKY + г nucleotide substitution model, strict molecular clock, and exponential growth coalescent model were used in the analyses. Independent runs were performed with a chain length of 200 million steps and sampled every 1,000 steps. Results from different runs were combined in Tracer v1.7.2 (43) to ensure an adequate effective sample size (>200) for relevant parameters.

Through phylogenetic analysis, 10 clusters were identified for the Omicron genomic sequences imported from other regions in China and local Omicron genomic sequences in Shandong. To depict genetic variation within the largest cluster, minimum spanning tree (MST) was generated based on the pairwise genome SNP distance using R package igraph. MST was visualized using R package visNetwork. To investigate the transmission patterns of two clusters that contained a mixture of domestic imported genomic sequences and local genomic sequences from Shandong, ancestral strait reconstruction was performed using BEAST v1.10.4 (40, 41). The analysis implemented an asymmetric substitution model and a strict clock model. Similar approaches for the molecular clock analysis were applied to ensure an adequate effective sample size (>200) for relevant parameters. Shandong Omicron genomic sequences were annotated as “introduction” or “local.” Local transmission lineages were defined as two or more Shandong genomes that descend from a shared introduction of the virus into Shandong. Introductions that lead to only one single case were referred to as “singletons.” The number of genomes in each local transmission lineage was inferred using R package NELSI (44).
